# A Critical Review for an Accurate Electrochemical Stability Window Measurement of Solid Polymer and Composite Electrolytes

**DOI:** 10.3390/ma14143840

**Published:** 2021-07-09

**Authors:** Adrien Méry, Steeve Rousselot, David Lepage, Mickaël Dollé

**Affiliations:** Département de Chimie, Université de Montréal, 1375 Avenue Thérèse-Lavoie-Roux, Montréal, QC H2V 0B3, Canada; adrien.mery@umontreal.ca (A.M.); steeve.rousselot@umontreal.ca (S.R.); david.lepage.3@umontreal.ca (D.L.)

**Keywords:** electrochemical stability window, solid polymer electrolyte, solid composite electrolyte

## Abstract

All-solid-state lithium batteries (ASSLB) are very promising for the future development of next generation lithium battery systems due to their increased energy density and improved safety. ASSLB employing Solid Polymer Electrolytes (SPE) and Solid Composite Electrolytes (SCE) in particular have attracted significant attention. Among the several expected requirements for a battery system (high ionic conductivity, safety, mechanical stability), increasing the energy density and the cycle life relies on the electrochemical stability window of the SPE or SCE. Most published works target the importance of ionic conductivity (undoubtedly a crucial parameter) and often identify the Electrochemical Stability Window (ESW) of the electrolyte as a secondary parameter. In this review, we first present a summary of recent publications on SPE and SCE with a particular focus on the analysis of their electrochemical stability. The goal of the second part is to propose a review of optimized and improved electrochemical methods, leading to a better understanding and a better evaluation of the ESW of the SPE and the SCE which is, once again, a critical parameter for high stability and high performance ASSLB applications.

## 1. Introduction

Li-ion batteries have been marketed since the early 1990s for several common applications, especially in portable devices and more recently for transportation and stationary power storage [1]. Compared with other energy storage devices, lithium-ion batteries have demonstrated many advantages including good energy density and long cycle life. However, even if a lot of progress has been made in recent years (for instance, efficient electric vehicles are presently available on the market), commercial batteries still need to be improved to achieve high energy and power densities while complying with safety requirements [2]. These current limitations are partly due to the restrictions imposed by electrode materials but also due to the poor stability (thermal, chemical, electrochemical, etc.) of liquid electrolytes. Indeed, the typical mixture of liquid alkyl carbonate solvents and lithium salts present in traditional Li-ion batteries is highly flammable and may lead to critical safety issues [2,3]. Thus, improving the safety of Lithium batteries is an important parameter that has concerned both the industrial and scientific communities for many years.

**Figure 1:**
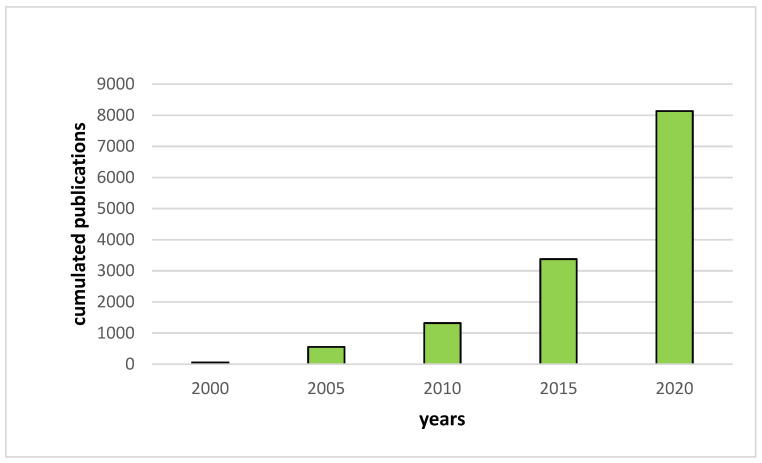
Evolution of the number of publications per year based on Web of Science results for “all solid-state battery”.

Thereby, the research on the development of new Lithium battery systems such as All Solid-State Lithium Batteries (ASSLB) has greatly increased since the early 2000s (Figure 1). In these devices, the traditional organic liquid electrolyte is replaced by a solid electrolyte that is both non-flammable and highly thermally stable. Furthermore, as no liquid is used in solid electrolytes, the entire battery packaging can be simplified, reducing dead weight, which results in an increase of the gravimetric and volumetric energy densities [4].

To date, three major categories of solid electrolytes (SE) have been developed: Inorganic Solid Electrolytes (ISE), Solid Polymer Electrolytes (SPE) and Solid Composite Electrolytes (SCE) [5]. ISEs generally have better ionic conductivities and lithium transference numbers than SPEs, but the latter present more flexibility, better processability and generally a good interfacial contact with the electrode materials, whether it is lithium metal or a composite electrode [6]. SCEs, which are a mix of ISE and SPE, tend to present the best properties of both SPEs and SCEs. Solid electrolytes must respect specific requirements:

- possess high ionic conductivities (around 10^−3^–10^−4^ S/cm at room temperature);
- negligible electronic conductivity;
- have high ionic transference number;
- have high mechanical and chemical stability;
- and possess a high Electrochemical Stability Window (ESW).

Most publications mainly focus on the ionic conductivities and transport properties of electrolytes before implementing them in full systems (usually using LiFePO_4_ (LFP), LiMn_2_O_4_ (LMO) or LiNi_x_Mn_y_Co_z_O_2_ (NMC) as a positive electrode and Li, Li-In or Li_4_Ti_5_O_12_ as a negative electrode). One of the current objectives for ASSLBs, and consequently the aim of the solid electrolyte, is to be able to operate at a high potential with high voltage cathode materials (NMC, NCA) working above 4.2/4.3 V vs. Li^+^/Li while being compatible with metallic lithium at the negative electrode. It is worth noting that there is a significant difference between being stable over one cycle (for example linear sweep voltammetry experiments), being stable for a few hundred cycles (what most of the publications report) and for thousands of cycles (industrial requirement). One way to predict the stability of the system during cycling is by analyzing the coulombic efficiency (CE). However, the fading of CE during cycling is rarely well understood and can be attributed to many factors such as the formation of dead Li, a bad cell assembly, and also to the degradation of the electrolyte. Thus, accurately assessing the stability of the electrolyte is one of the significant parameters that must be addressed due to the critical role of the ESW in the energy density and durability of the entire ASSLB. However, if the standardization of ionic conductivity measurements starts to appear [7], ESW, while recognized as a parameter of importance, comes almost secondary when compared to ionic conductivity. Thus, it is often inconsistently reported, determined, or evaluated.

Considering that an accurate measurement of the electrochemical stability window is a key parameter for the development of all solid-state batteries, we propose in this work a literature overview that focuses specifically on the evaluation of the electrochemical stabilities of Solid Polymer Electrolytes (SPEs) and their composites with ceramic/inorganic fillers (SCEs).

In the first part, an overview of recent articles is presented via a table regrouping several characteristics of SPEs and SCEs. This is paired with a description of traditional ways that are used by a large part of the ASSLB community to evaluate the ESW for electrolytes.

The second part is dedicated to upgraded methods and practices for a careful evaluation of ESWs. Additionally, we will present our vision on how to accurately measure the ESW of an electrolyte. The objective is to highlight the best measurement techniques to generate a better understanding and a more realistic interpretation of ESW values for SPEs and SCEs, which are expected to lead to better ASSLB stability and performance.

## 2. ESW Evaluation: Overview of the Recent Literature on SPE and SCE with Typical ESW Measurements by LSV/CV Methods

This section focuses on the ESW evaluation for SPEs and their composite counterparts with ceramic/inorganic fillers (SCE). Typically, am SPE consists of a polymer matrix with an alkali metal salt, i.e., lithium salt for ASSLB applications. In addition to the previously mentioned properties, the SPEs are expected to reduce/prevent Li dendrite formation and decrease the electrode/electrolyte interface impedance thanks to an optimal contact [8,9].

One of the most studied SPEs is polyethylene oxide (PEO), a polyether which attracted a lot of attention following the first reports describing its ionic conductivity in the presence of alkali metal salts [10,11,12]. Since then, PEO-based electrolytes have undergone a lot of development and several studies based on PEO and PEO derivatives have been conducted [13,14,15,16]. Throughout the years, research on non-polyether-based SPEs (alone or mixed/grafted with PEO) and composite electrolytes have been performed [16,17,18,19] to overcome limitations in the ionic conductivity and electrochemical stability of PEO. SCEs are generally prepared by the dispersion of inorganic fillers in the polymer matrix to enhance the mechanical properties, the interfacial stability/compatibility, and the ionic conductivity (by decreasing the crystallinity of the polymer host) of the solid composite electrolyte.

Table 1 groups several results including values of E_ox_ for SPEs and SCEs (depending on the method of evaluation, temperature of the experiment, and scanning rate during the analysis) from recent articles spanning 2019–2020. It is worth noting that ESW includes stabilities in terms of oxidation (E_ox_) and reduction (E_red_) potentials. When an additional current to the capacitive one appears, this underlines a faradic reaction (oxidation or reduction of the material/electrolyte) beyond which the system is no more stable. Some authors make the distinction between E_ox_ and E_red_. However, in most papers the study is only concentrated on E_ox_ because most SPEs and SCEs are assumed to be compatible with Li metal (specifically those containing PEO). Thus, ESW is sometimes simply assimilated to E_ox_.

Several observations can be made based on the data presented in Table 1. First, based on these recent works, we can see that PEO is still one of the most investigated polymers. However, as discussed above, polymers are now rarely used alone, but are instead blended with other polymers, copolymerized, and/or filled by ceramic/inorganic fillers (Metal Organic Frameworks (MOFs), SiO_2_, BaTiO_3_, MnO_2_). Another point is that even if some authors try to use new lithium salts, LiClO_4_ and LiTFSI are still the most popular salts for SPE and SCE applications.

This overview of SPEs and SCEs based on recent literature allows us to evidence not only the variety of the tested systems but also of the experimental parameters (method, scanning rate, temperature) for the ESW evaluation of the solid polymer and composite electrolytes. Also, upon scanning the literature, the ESW is recognized as a parameter of importance but appears to come almost secondary in comparison to ionic conductivity. Consequently, the accurate evaluation of the electrochemical stability (ESW) of the electrolytes is often briefly addressed in most papers.

**Figure 2:**
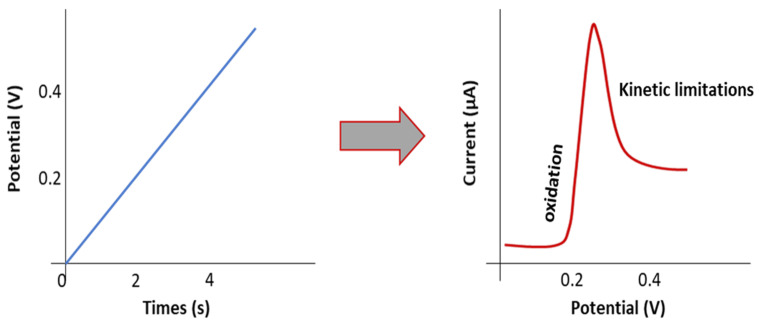
Scheme representing the principle of the LSV method.

**Figure 3:**
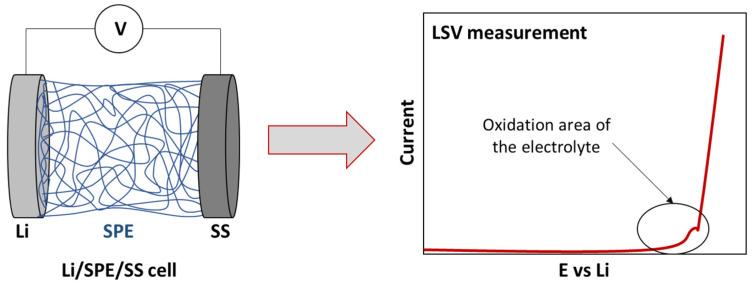
Scheme of traditional cell for ESW evaluation by LSV measurement.

ESW is usually evaluated by the Linear Sweep Voltammetry (LSV) method in a lithium/electrolyte/inert planar electrode (Pt, Stainless steel) cell configuration. Inert working electrodes are used to avoid parasitic reactions and interfering current backgrounds (Figure 2 and Figure 3). Typically, the current at a working electrode is measured while the potential is swept linearly vs. time from lower to higher potentials.

**Figure 4:**
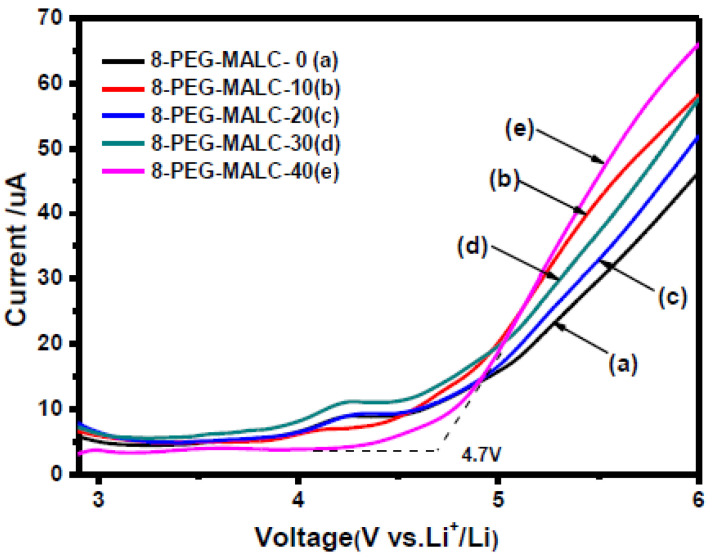
Linear sweep voltammetry of different liquid crystalline copolymer composite polymer electrolytes at 0.1 mV/s with a cut-off limit evaluated at 4.7 V; extract from ref. [38].

The objective is to detect at which potential the SPE or the SCE will be oxidized/reduced and, thus, degraded. Basically, when LSV is applied, after the capacitive current associated with the electrochemical double layer (which appears as soon as the potential of the electrode is changed), the oxidation (faradic current) is determined when a sudden increase of the current appears at a given potential. Figure 4 shows an example of an experimental LSV profile. Depending on the authors, experimental parameters such as temperature evaluation, scanning rate, determination of the potential estimated as the beginning of the oxidation (4.7 V in this example), or the minimum current value considered as a realistic value meaning that oxidation occurs, are quite different. We need to keep in mind the non-ideal conditions when performing ESW assessment for SPEs or SCEs. An analogy with liquid electrolytes can be demonstrated. In liquid electrolytes, new species diffuse from the bulk towards the electrode. New oxidizable species will diffuse and it will become complicated to get practical limiting current values. Now for the polymeric systems, another limitation appears. Indeed, they do not diffuse, or hardly diffuse, which means no renewal of the oxidizable species is possible. This places emphasis on the amount of SPEs initially in contact with the electrode. These limitations lead to the necessity of having a large analysis area for the ESW determination of SPEs and SCEs. Thus, it is hard to point out precise values and a standard ESW evaluation method.

**Figure 5:**
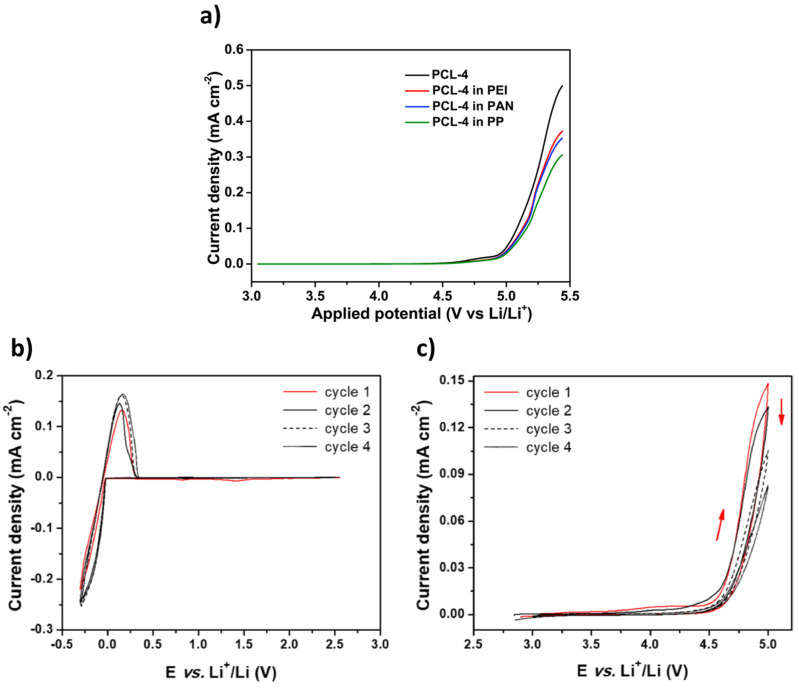
(**a**) Example of LSV profiles at 1 mV/s and 55 °C. Extracted with permission from ref. [17]. Copyright 2020 Elsevier. (**b**) and (**c**) CV profiles at 60 °C and a scanning rate of 0.1 mV/s; (**b**) cathodic stability highlighting the Lithium oxidation/reduction at low potentials (cell configuration: Li/SCE/Cu); and (**c**) anodic stability highlighting the oxidation of the electrolyte at high potentials (cell configuration: Li/SCE/Carbon black). Extracted with permission from ref. [62]. Copyright 2019 Elsevier.

**Figure 6:**
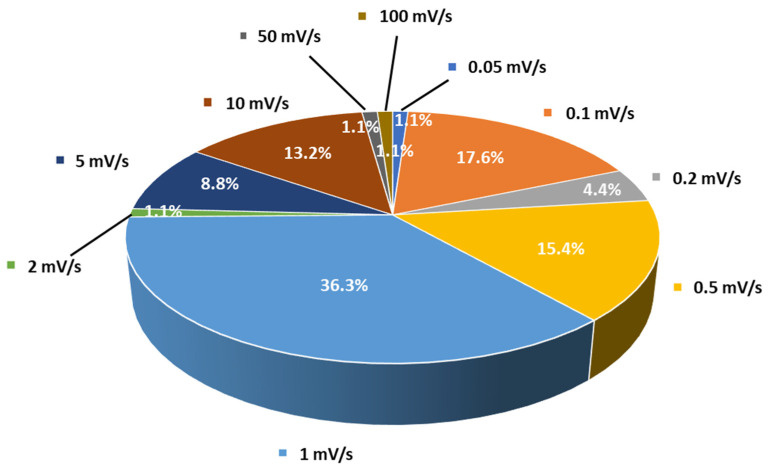
Distribution of scanning rates (%) used by authors for ESW evaluations taken from Table 1.

Cyclic voltammetry is the second most frequently used method for evaluating the ESW of SPEs or SCEs. In a typical experiment, the working electrode potential is ramped linearly versus time from the open circuit voltage to a set potential. After the set potential is reached, the working electrode’s potential is ramped back in the opposite direction to the initial potential. These cycles of potential sweeps can be repeated as many times as needed (Figure 5b,c). However, it is important to keep in mind that once the first oxidation is initiated, it is likely that during the other cycles the material has been altered due to this first oxidation. The current at the working electrode is plotted versus the applied voltage to give the cyclic voltammogram. Oxidation (positive current) and reduction (negative current) peaks can be observed if redox species react at the surface of the working electrode. These reactions can be related to the stability of the species. Especially, when analyzing electrolyte solutions and naturally for solid electrolytes such as SPEs or SCEs, the appearance of intense redox peaks at a given potential is characteristic of the occurrence of a reaction which can be related to electrolyte degradation. It is important to note that two different cells must be used for the evaluation of the anodic and the cathodic stability of the electrolyte even if some authors still use the same cell for both analyses. For instance, in their work, Piana et al. [62] made the choice to evaluate the SCE (PEO/LAGP hybrid electrolyte) by scanning the cell potential from the OCV (open circuit potential) down toward −0.3 vs. Li^+^/Li and, then, upward to 2.5 V vs. Li^+^/Li (cathodic stability window) in a Li/SCE/Cu cell configuration, and from the OCV toward 5.0 V vs. Li^+^/Li and, then, downward to 2.5 V vs. Li^+^/Li (anodic stability window) in a Li/SCE/Carbon black cell configuration with a scanning rate of 0.1 mV/s. In that case, both cells use planar working electrodes (Cu and carbon black coated Al foil) for the ESW evaluation. Thus, the stability of the SPEs and SCEs can be analyzed via CV in a similar way to with LSV. The cell configuration is often identical to the one for LSV analysis and, once again, a huge range of choices about the setup of parameters like temperature and scanning rates is also observed. For instance, Figure 6 shows the distribution of scanning rates used for ESW evaluation by LSV/CV taken from Table 1. A clear trend towards low and moderate scanning rates (0.1 to 1 mV/s) is observed. However, many other authors use higher scanning rates (>5 mV/s). It must also be noted that even 1 mV/s can be considered to be a high scanning rate for ESW evaluations because of the sluggish processes involved in the degradation of solid-state electrolytes.

Thus, it is hard to conclude how representative these tests are of the true ESW. No strict rules are established for evaluating the ESW, even if low scanning rates seem like they would be beneficial, and it is difficult to get a satisfying conclusion about the realistic impact of these values [114]. Other factors such as how long the system experiences the potential, the analysis volume, and the non-mobility of the polymer (for SPE) must be addressed. Nevertheless, this methodology has changed little over time [115,116,117,118].

## 3. Toward a More Specific and Better Evaluation of the ESW

For ASSLB, the use of solid electrolytes requires them to be stable at high potentials vs Li^+^/Li and compatible with high voltage cathode materials (NMC, NCA) operating up to 4.2/4.3 V [96,119,120] while remaining compatible with the lithium metal anode. Surprisingly, despite high oxidation limits claimed in most publications (largely above 4.0 V vs. Li^+^/Li), the electrochemical characterization of these materials in a battery set up is usually performed using LFP cathodes, which have a redox potential of 3.45 V vs. Li^+^/Li. The fact that the well-known cathode material NMC, which has a potential window of 3.0–4.3 V vs. Li^+^/Li, is not used in these publications to further qualify the stability of their electrolyte leads to questions regarding the validity of the ESW measurement.

The purpose of this section is to highlight articles that are specifically dedicated to the evaluation of the ESW of electrolytes. This chapter is focused on describing “advanced” methods to accurately evaluate the ESW of SPEs and SCEs. Some papers on Inorganic Solid Electrolytes (ISE) or liquid electrolytes are also addressed in this part when an interesting approach to the ESW evaluation is conducted and that can be adapted for use in systems employing SPEs and SCEs.

Even if it is barely addressed, some authors try to improve the evaluation of the electrochemical stability of the electrolytes. Two main characteristics arise regarding a suitable evaluation of the ESW: the cell construction and the optimization of the experimental setup generally based on the techniques previously described (LSV and CV).

The architecture and the configuration of the electrochemical cell for ESW determination is indeed an important parameter. Although standard cells consist of three-electrodes (working electrode, counter electrode and reference electrode), most of the measurements, for convenience, (specifically for battery applications) are made in a two-electrode cell configuration (with one electrode playing the role of both reference and counter electrode). Most of the studies use metallic lithium as both reference and counter electrode assuming that the SPE or SCE materials are stable vs. lithium or indium-lithium alloys and stainless steel as a working electrode. Sometimes these experimental considerations vary (different electrode materials) and strongly impact the value of the ESW. These considerations will be discussed in detail in the next section.

### 3.1. Cell Configuration

As was mentioned previously, the standard cell configuration for the evaluation of the ESW is a Lithium/electrolyte/stainless steel (SS) cell (Figure 3). Sometimes, variations, such as the use of other inert planar electrodes, can be observed. For instance, Piana et al. [62] used Cu metal foil and carbon black coated Al foil as working electrodes for cathodic and anodic scans, respectively, with Li metal as both the counter and the reference electrodes. However, some authors who have focused on characterizing suitable ESW evaluation insist on the importance of the cell configuration and especially on the suppression of the inert planar electrode. Indeed, their geometric surface area and chemical composition is negligible compared to that of the composite electrodes that are used in practical battery devices and, thus, disregard the real electrochemical environment of the evaluated electrolytes. This often results to an overestimation and inappropriate ESW values.

In their work (1999) Xu et al. [121] recommended that to achieve a real electrochemical stability window (for capacitor and battery applications), the electrode material used for the ESW evaluation should simulate the electrodes used in a real system. The electrolyte stability data generated by conventional approaches (cf. Section 1) could be inaccurate when applied to electrolytes in real devices. Although this work dealt with liquid electrolytes, it is reasonable to assume that these ideas could be extended to solid electrolytes (i.e., with SPEs and SCEs). Some experimental details of their work are presented here. First, the electrolytes, electrode materials, and subsequent measurements were all handled under vacuum in an argon-filled glove box where both H_2_O and O_2_ content was below 5 ppm. Solvents of electrolytes were redistilled and well dried until the moisture content decreased below 100 ppm (around 50 ppm as determined according to the Karl Fischer titration method).

Linear or cyclic voltammetry were used for measuring the current-potential (i-V) polarization curves of the tested electrolytes. The three-electrode cell configuration was employed with either Pt wire, glassy carbon (GC), activated carbon (AC) film or lithium battery cathode composite Li_x_Mn_2_O_4_ as working electrodes, and with Li^+^/Li as the reference electrode. Lithium foil was used as the counter electrode for lithium battery tests. A scan rate of 5.0 mV/s was typically used, except for cathode composite where the measurement was performed at 0.1 mV/s, owing to the slow lithium-ion diffusion process inside the spinel materials.

First, they applied the conventional approach (i.e., linear or cyclic voltammetry on nonporous electrode is used for the determination of the ESW). As they explained, the limiting redox potentials are ascribed where the de-composition current achieves a predefined level. However, the choice of these cutoff criteria is not supported by any theoretical considerations and is therefore arbitrary. Depending on the authors, cutoff currents of 10 µA/cm^2^, 50 µA/cm^2^, 0.5 or 1 mA/cm^2^ (most popular) can be chosen. At the end, the difference in stability data imposed by these arbitrary cutoff criteria would be too important to ignore and could lead to conflicting conclusions on the electrolyte stability evaluation.

Moreover, authors claimed that the difference between cutoff currents is not the only reason for the inaccuracy of conventional ESW evaluation methods. To satisfy this conventional method, they identified two prerequisites. First of all, the capacitance of the working electrode must be negligible. Then, the faradic component of the current (I_f_) must only be dictated by a decomposition mechanism. These settled conditions imply that no other faradic process than electrolyte decomposition takes place and can be sharply approached when nonporous electrodes such as GC or Pt are used in a potentiodynamic experiment due to their stability. This allows the absence of other faradaic processes on the working electrode, except for impurities which can be easily lowered beneath 0.1 mA/cm^2^ by rigorous drying of the electrolyte solvent and high purification of the electrolyte solution. However, these ideal conditions are no longer valid for battery applications, where electrode materials are not GC or Pt but rather various composite materials with high capacity which results in additional faradic processes. Furthermore, as active material are often moderate electronic conductors, conductive additives (usually carbon black) are added to the electrode formulation. Thus, the non-faradic part of the current density is no longer negligible as in the ideal case mentioned above. Also, the electrolyte decomposition is affected by the surface of the composite electrode. In conclusion, a narrower ESW should result for real systems and results obtained by conventional analyses with GC or Pt working electrodes cannot describe the anodic stability of electrolytes in real battery devices. Thus, according to the authors, using the same electrode material as that employed with the electrolyte in the real battery cell is the only way to obtain a reliable evaluation of the electrochemical stability window.

About their experimental results, they concluded on the inaccuracy of the stability window measurement when employing nonporous electrodes on the battery electrolyte. They determined that using the electrode material approaching the surface state and surface area of the electrodes used in real devices was the best solution. Authors claim that this solution should be universal, instead of being considered confined to the determination of anodic stability of battery electrolytes only. They also concluded about the fact that few ESW values had been obtained in this way at the time of their article. “That time” was in 1999 and, considering that the first part of this review was only based on recent papers (2019–2020), it seems that twenty years did not make a significant difference.

Other works insist on the use of working electrodes that are as close to the real battery electrodes as possible for ESW investigations. Kasnatscheew et al. performed a study [122] in which they argue that even though a classical measurement (LSV) with an inert working electrode (Pt, glassy carbon) is well adapted for qualitative comparison, it becomes inappropriate in the prediction of a precise electrochemical stability value. This is mainly attributed to the electrode surface area, which is different from that of high surface composite electrodes used in practical devices. This affects the current density and, consequently, the overpotential. Additionally, the electrode composition and surface area are presumed to have an impact on the catalytic activity. To overcome these drawbacks, ESW measurements with LMO electrodes are proposed. This condition reasonably reflects a more realistic battery application. However, concerns and issues linked to this method are still pointed out by authors. There are doubts concerning the validity of this method, as it is not clear whether the obtained stability data can actually be transferred to a real LIB device. First, the chemical composition of the different active materials used in LIBs can, for instance, affect the stability limit via catalytic effects. Then, the determination of the ESW is based on a potentiodynamic principle, while LIB cycling is based on a constant current principle. These differences could have an influence on the determination of the electrolyte stability limit.

### 3.2. Other Methods: Improved Setups for the ESW Evaluation

In addition to the cell construction and the importance of using composite working electrodes, some authors focus on improved ways to use LSV or CV by adjusting parameters (sometimes articles still mention the use of inert working electrodes but with improved LSV or CV set up).

Hallinan et al. [123] proposed an electrochemical approach based on a series of adjusted LSV measurements from different, large over potentials to open circuit voltage, which the authors name “variable reverse linear sweep voltammetry” for evaluating the ESW of solid polymer electrolytes. By applying relaxation times to the cell between each polarization, the first data points of each voltammogram are not limited by mass transfer. This allows the current vs overpotential data to be analyzed by a kinetic model such as the Butler–Volmer one.

Electrochemical measurements were conducted on PEO and SEO (Polystyrene–b–poly(ethyleneoxide)) containing LiTFSI salt in cells with different working electrode materials in both two and three electrode cell configurations at a scanning rate of 15 mV/s. The electrode materials are either used as current collectors in lithium-ion batteries or are intended as inert electrodes for examining oxidative degradation of the polymer electrolytes. An electrochemical impedance spectroscopy (EIS) measurement was performed for every sample before and after every set of electrochemical tests to ensure that the sample was still exhibiting behavior in the same way as a pristine cell. Among the results obtained by these upgraded LSV measurements, the authors found an electrochemical stability for the SEO electrolyte of around 5 V at 40 °C. Based on their experiments on Cu/SEO/Li cells, they concluded that over-discharge should be avoided to prevent Cu corrosion (as with liquid electrolytes for battery applications). This work demonstrated a different way of using LSV methods to improve precision in the determination of the ESW.

Kasnatscheew et al. [122] assessed the validity of the potentiodynamic based ESW method by comparing the data with that obtained by galvanostatic method on commercial positive electrodes. They demonstrated the good agreement of the two methods on the determination of the oxidation stabilities of electrolytes. Additionally, they were able to quantify the parasitic reactions by comparing the specific capacity losses obtained in half of the cells during cycling experiments.

Even though this work is devoted to liquid electrolytes, it highlights the use of composite battery electrodes to obtain a more realistic evaluation of the ESW of the electrolyte, and these observations can be transferred to SPEs and SCEs.

**Figure 7:**
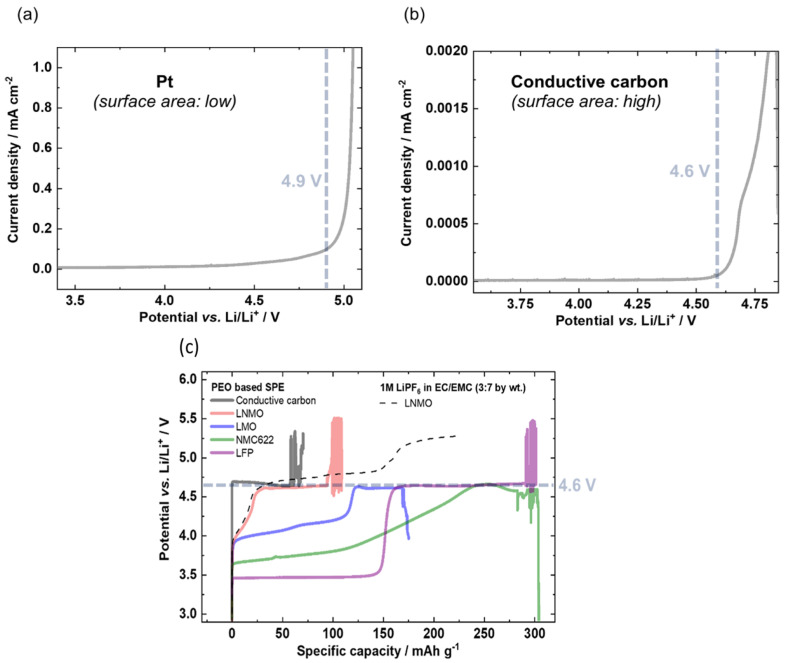
LSVs of PEO based SPE with a scan rate of 0.1 mV/s on (**a**) Pt and (**b**) conductive carbon working electrode resulting in an exponential increase in current density of 4.9 and 4.6 V vs. Li/Li^+^, respectively. (**c**) Determination of the onset of main oxidation of PEO based SPEs via overcharge of the working electrode with a specific current of 15 mA g^−1^ using different positive electrodes. Extract with permission from ref. [124]. Copyright 2020 Springer Nature.

A similarly comparative study based on potentiodynamic and galvanostatic results for several active materials was conducted by Homann et al. [124]. In their work, they consider PEO-based SPE under battery cell operation to evidence cell failure and to clear up reported ambiguities regarding oxidation stability. Then, they conducted electrochemical stability evaluations on various cathode materials that are usually used in Lithium batteries (NMC, LMO, LNMO and LFP) via LSV (potentiodynamic) and galvanostatic measurements. The onset of oxidation can be detected by an exponential (e.g., Butler–Volmer) current increase and a potential plateau, respectively. LSV measurements were conducted with an applied scanning rate of 0.1 mV/s. For the galvanostatic approach, electrodes were charged with a specific current of 15 mA/g. Preliminary evaluations were conducted by LSV on a Pt inert working electrode and revealed an Eox around 4.9 V (the choice of the cut-off current was fixed arbitrary without further explanation). However, as mentioned earlier in this review and by the present authors, the validity of this result is questionable, as the surface area of the Pt foil is small compared to that of composite battery electrodes in practical cells. Then, they decided to implement a galvanostatic approach to improve the accuracy of their measurement. Thus, LSV on a conductive carbon electrode with higher surface area was applied and a lower onset oxidative potential of 4.6 V was obtained. Moreover, to be closer to real evaluating characterizations and battery conditions, galvanostatic experiments were performed on a conductive carbon electrode, NMC, LNMO, LMO and LFP working electrodes to confirm the LSV observations. Like the LSV experiment, the galvanostatic approach revealed that the onset of oxidation occurred at 4.6 V vs. Li^+^/Li, as seen by the respective potential plateau. These results are shown in Figure 7.

In this study, the authors also concluded that the main source of the sudden battery failure was the Li/SPE interface and, particularly, Li dendrite formation and penetration through the SPE membrane rather than the SPE/NMC interface. Finally, they claim that “it is the cell set-up (PEO thickness, negative electrode), which is crucial for the voltage-noise associated failure, and counterintuitively not the high potential of the positive electrode.”

**Figure 8:**
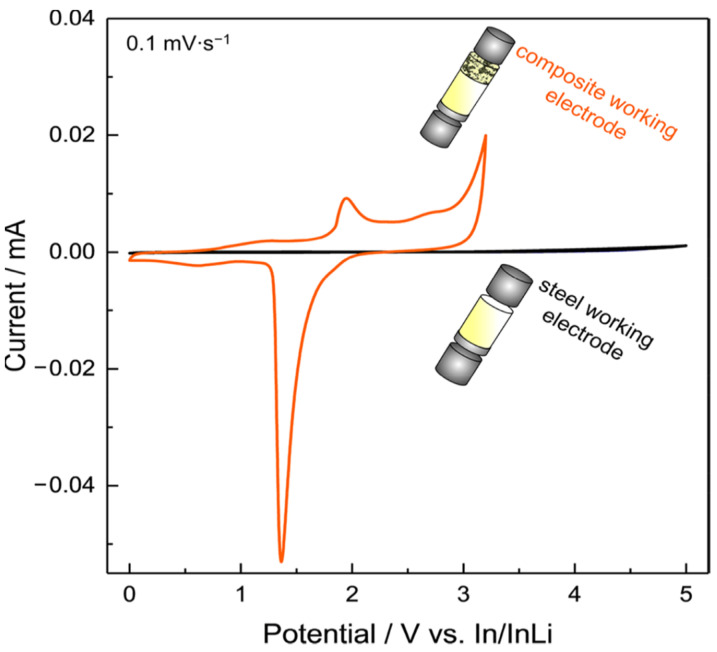
Visual comparison of two types of electrode morphology in CV experiments with the thiophosphate solid electrolyte Li_10_GeP_2_S_12_. Reprinted with permission from ref. [125]. Copyright 2019 American Chemical Society.

Another interesting work proposes an improved CV setup to analyze the ESW of a solid electrolyte [125]. Dewald et al. applied what they call a “stepwise cyclic voltammetry” method to evaluate the practical oxidative stability of various inorganic solid electrolytes (SE) such as Li_10_GeP_2_S_12_, Li_2_S-P_2_S_5_ or Li_6_PS_5_Cl. For reasons already mentioned in the previous section about the necessity of avoiding the use of planar electrodes, the authors decided to replace the traditional working electrode by a SE-carbon black composite electrode with higher surface area in order to increase the interfacial contacts between the components and, thus, the sensitivity of the measurement. Additionally, as the electrolytes are expected to decompose in contact with Li metal, indium metal is used as a counter electrode to minimize the reaction current arising from the decomposition on the anode side and to ensure that no additional lithium source is present. Their results, presented in Figure 8, clearly show the impact of the replacement of the inert working electrode by a composite electrode on the ESW determination.

By employing In/InLi as both the reference/counter electrode and planar stainless steel as the working electrode, only small currents are detected (black curve). Using a carbon−solid electrolyte composite electrode to the cell (orange curve) leads to higher currents. Consequently, important oxidative decomposition reactions are now visible, mainly due to the huge difference in surface area between a flat (steel) and 3D conductive electrode (composite carbon electrode). In order to determine more precisely the oxidation onset potential of the electrolyte they established a stepwise CV approach. Each CV was measured twice at a low scan rate (0.1 mV/s) followed by a stepwise increase of the potential range by 0.1 V up to 4.4 V vs. In/InLi (approx. 5 V vs. Li^+^/Li). Authors once again pointed out the necessity of avoiding the use of classical working planar electrodes (SS, Pt) for a better evaluation of the ESW. Their stepwise cyclic voltammetry method could be transferred to the evaluation of SPEs and SCEs. Other works propose the use of solid electrolyte composite electrodes for better ESW evaluations with carbon [126]. It has to be noted that the use of carbon for such measurement could be biased by the presence of residual water within the carbon. Careful drying of carbon is therefore required [125]. Other authors will then prefer gold to carbon for instance [127].

**Figure 9:**
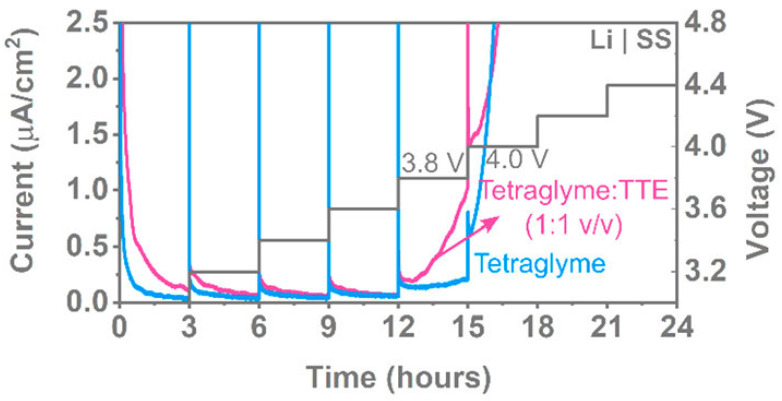
Potentiostatic holds for 3 h at different voltages with stainless steel as the working electrode and 0.1 M LiFSA in tetraglyme and tetraglyme:TTE. Reprinted with permission from ref. [128]. Copyright 2020 American Chemical Society.

Lastly, Amanchukwu et al. [128] synthesized a new class of fluorinated ether electrolytes that combine the oxidative stability of hydrofluoroethers (HFEs) with the ionic conductivity of ethers in a single compound. Among their main results, they showed that their fluorinated ether electrolytes can achieve an ionic conductivity of 2.7 × 10^−4^ S/cm at 30 °C with a higher oxidative stability of up to 5.6 V compared to classical ether electrolytes. They used two methods to determine the oxidative stability of the electrolytes. In the first approach they used a classical LSV measurement in a SS/Li cell with an applied scanning rate of 0.1 mV/s from an open circuit to 6 V. They observed good electrochemical stabilities up to 5 V. They also used potentiostatic holds (Figure 9), also called Potentiostatic Intermittent Titration Technique (PITT), to accurately probe the oxidative stability of the fluorinated ether electrolytes. They argue that long Potentiostatic hold experiments are less sensitive to the influence of impurities which could lead to an early increase of the current in LSV experiments not corresponding to the actual oxidation of the compound. Finally, their conclusions were made by using a stainless steel, aluminum, or Ni-rich NMC 811 electrodes with different electrolytes and by holding the potential for 3 h at increasingly higher potentials. The recorded current should decrease if no undesired Faradaic reactions occurs. Their best synthesized fluorinated ether electrolyte reached an oxidative stability of 5.6 V. Despite, the fact that this study was conducted on liquid electrolytes, the electrochemical “potentiostatic holds” method could serve as a solid base and could be extended to the ESW evaluation of Solid polymer electrolytes. For instance, Zhang et al. [129] used this method of potentiostatic holds to determine the anodic stability of different polymer electrolytes.

**Figure 10:**
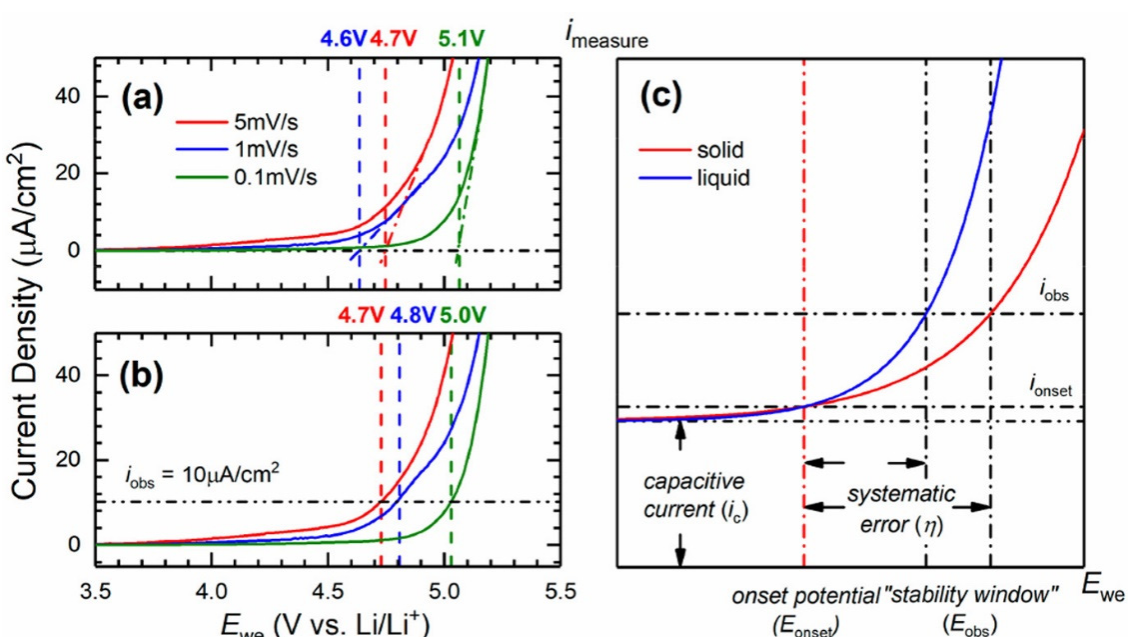
Linear sweep voltammogram of PEO:LiTFSI (O/Li = 10) from 3.5 to 5.5 V vs. Li^+^/Li at room temperature. Examples of “stability thresholds” determined by (**a**) extrapolating the voltammogram and (**b**) using an arbitrary onset current density. (**c**) Description of the systematic error in estimating the oxidative stability from current-based methods. Reprinted with permission from ref. [130]. Copyright 2021 American Chemical Society.

Recently, Li et al. [130] determined the absolute anodic stability threshold of polymer electrolytes via a capacity-based electrochemical method. The objective was to address the limitations imposed by traditional approaches such as LSV. The authors claim that “the inconsistency of LSV is intrinsic to the method”. The comparison of electrolytes with distinct conductivities is problematic due to the proportionality of the current density and the electrolyte conductivity. Mass transport limitations cause difficulty in the theoretical analysis and lead to greater error for any method based on current density. Their point is clearly presented in Figure 10.

The authors explain that close to the true stability potential of the electrolyte (E_onset_), the Faradaic current (i_onset_) and the capacitive current (i_C_) may be too intricate to be distinguished. Thus, to clearly assign a faradic reaction, a significant increase of the observed current density is needed and the systematic error is the difference between i_onset_ and i_obs_. Due to the slow kinetics and low diffusion processes in solid state systems, the i-E curve will be flattened, and a larger error is expected compared to liquid electrolytes. Also, the shape of the voltammogramm can influence the value of the systematic error which becomes unique to each case and quasi-impossible to correct between samples or by repeated measurements. Thus, a noticeable disparity is expected to be inherent to the LSV method for the ESW determination of polymer electrolytes.

Thus, in order to overcome these limitations, they developed an alternative method based on capacity measurements which they named the reversibility test. Briefly, a cyclic voltammetry is performed, and the charge capacities of the cathodic and anodic responses are compared. The ratio between the anodic and the cathodic capacity corresponds to the irreversibility of the process. The capacity ratio remains similar when the electrolyte is electrochemically stable but varies when the potentials exceed the stability threshold of the electrolyte. By this method, the anodic stabilities of poly(ethylene oxide) (PEO) and hydrogenated nitrile butadiene rubber (HNBR), both blended with lithium bis(trifluoromethanesulfonyl) imide (LiTFSI), were identified to be 3.6 and 3.7 V vs. Li^+^/Li, respectively.

Finally, other studies use computational methods (as DFT calculations) to determine the electrochemical stability of liquid and solid electrolytes [131,132] or a complementary study involving both experimental and computational methods [126,127,133,134,135]. In their work, Thompson et al. [134] proposed a complementary computational/experimental work by using alternating current electrochemical impedance spectroscopy, direct current chronoamperometry, and optical absorption band gap measurements combined with first-principles calculations to characterize the electrochemical window of the Li_7_La_3_Zr_2_O_12_ (LLZO) solid electrolyte. These first-principles calculations were used to predict the density of states (DOS), band gap, and absolute positions of the band edges for LLZO. Authors employed three different levels of theory: (1) the semi-local generalized gradient approximation (DFT-GGA) of Perdew, Burke, and Ernzerhof (PBE); (2) the hybrid functional of Heyd, Scuseria, and Ernzerhof (HSE06); and (3) quasi-particle (QP) calculations based on many-body perturbation theory (G0W0 method). These methods were already used to predict the ESW of liquid electrolytes at electrode interfaces. This work is one of the good examples of the complementarity between experimental and computational methods that can improve the accuracy of the ESW evaluation of electrolytes including SPEs and SCEs.

### 3.3. Final Validation Tests

Ultimately, the objective of the present manuscript concerns not only the intrinsic stability of the electrolyte but also the electrochemical stability of the entire battery cell. As discussed before, in addition to the use of improved setups, performing tests with real battery electrode materials (cf. Section 3.1) in full cell configuration is critical for the validation of the overall electrochemical stability of the device. In such cases, the stability of the cell is diagnosed via the monitoring of the Coulombic efficiency and capacity retention. A Coulombic efficiency of at least >99.9% upon prolonged 99.9% is targeted to achieve high stability [136]. However, the difficulty of building an optimized and operating battery cell must be kept in mind. Assembling such cell implies the selection of various materials including the current collector, the choice and quality of the active materials and metallic lithium [137,138], and the optimisation of a considerable amount of parameters such as the composite electrode formulation [139], the electrodes and solid electrolyte thickness, the crystallinity (single/poly) of the cathode material, the issues associated with the use of metallic lithium [137] and, lastly, interfacial considerations [140,141] and other external controls (cycling current, pressure) [142].

## 4. Summary and Outlook

Based on the experiments that have been discussed in the previous section, general remarks and conclusions can be made.

First, the traditional use of LSV in a Li/electrolyte/SS cell seems to be acceptable for a qualitative approach but not accurate enough for a precise ESW evaluation. The limiting reduction and oxidation potentials are detected where the de-composition current reaches a predefined value. However, the choice of these cutoff criteria has no theoretical significance and is therefore completely arbitrary. Indeed, it can be tricky to discriminate the capacitive current from the faradic current which will depend of the studied material/electrolyte, the architecture of the working electrode (flat vs. 3D) and its type (carbon vs. Pt, Au…). Thus, depending on the authors, arbitrary cutoff currents of 10 µA/cm^2^, 50 µA/cm^2^, 0.5, or 1 mA/cm^2^ are regularly chosen. Moreover, a large range of scanning rates (0.1 to 10–50 mV/s) is used during testing. The difference in stability data is too significant to ignore and can lead to conflicting conclusions (often an overestimated value) when evaluating ESW data.

Also, several authors agreed that it was necessary to avoid the use of planar inert electrodes (SS, Pt…) for the evaluation of the ESW. Indeed, the use of planar electrodes often leads to an overestimated ESW value due to the small surface area and, thus, diminished contact with electrolyte which affects the current density and influences the overpotential. Thus, the association between the classical LSV and cell configurations with planar electrodes generally leads to misestimated ESW values. It is very surprising that a lot of studies still employ this setup for the determination of the ESW.

Finally, to efficiently evaluate the ESW, the two major aspects that must be considered are (i) a suitable cell configuration and (ii) an appropriate method of evaluation.

In regard to point (i), it is clear that the evaluation of the ESW must be conducted on cells containing a composite working electrode to increase the interfacial contacts with the electrolyte, compared to the commonly used planar electrodes, and to better simulate the real electrolyte environment present in a battery cell. This can be a solid electrolyte/carbon composite electrode (assuming no interfacial reaction happens at the surface of CB due to surface groups and/or remaining water) or the electrode material really used in the battery cell (LFP, LMO…)

Concerning point (ii), upgraded methods such as improved potentiodynamic techniques (CV or LSV using improved setups), PITT-type evaluation with impedance measurements, or galvanostatic techniques must be preferentially used. Performing these experiments with low scanning rates is also an important kinetic factor with respect to the sluggish degradation reactions in the complete solid state. Also, the complementarity of computational methods is beneficial for a better clarification of the ESW.

Even if all the upgraded methods for ESW evaluation presented in this study are not always specifically developed for the analysis of solid electrolytes, it is evident that those methods can and should be extended to solid-state systems for an accurate determination of the ESW of Solid Polymer Electrolytes and Solid Composites Electrolytes.

Also, we should pay more attention to the formation of the electrolyte degradation products. A better identification and characterization of such products could be the key to define precisely the ESW, to avoid their formation, to eliminate them, or eventually to limit their formation.

Finally, after a precise evaluation of the ESW of the electrolyte itself, the ultimate stability evaluation must be performed in full battery cells while keeping in mind the variety of parameters/components to optimize inside the cell. Currently, this is still a huge challenge to overcome for the future development of all solid-state lithium batteries.

## Figures and Tables

**Table 1:** Properties of various Solid Polymer-based electrolytes (PEO, other polymers and composites) from recent publications (2019–2020). The several reported properties are the ionic conductivity, the conductivity temperature evaluation, E_ox_ (oxidative stability of studied materials), the scanning rate for the E_ox_ evaluation, the temperature for ESW evaluation (RT = Room temperature) and the method used for the evaluation (cyclic voltammetry, CV; Linear sweep voltammetry, LSV).

Solid Electrolyte/Sample	Salt	Ionic Conductivity (S/cm)	Conductivity T (°C)	E_ox_ (V vs. Li^+^/Li)	Scan Rate (mV/s)	ESW T (°C)	Method	References
PCL	LiTFSI	2.5 × 10^−5^	RT	4.6	1	55	LSV	[17]
PIL-SN-PCE	LiTFSI	6.54 × 10^−4^	RT	5.4	1	/	LSV	[18]
DAVA + ETTMP 1300	LiPF_6_	7.65 × 10^−4^	RT	6	100	/	LSV	[19]
PEO8–LiPCSI	LiPCSI	7.33 × 10^−5^	60	5.53	0.2	60	LSV	[20]
3D ANF framework/PEO-LiTFSI	LiTFSI	8.8 × 10^−5^	RT	>4.5	1	/	LSV	[21]
PEO–LiClO_4_–LLZTO	LiClO_4_	/	60	4 to 4.5	0.3	/	LSV	[22]
PEO-LiTFSI-3%VSB-5	LiTFSI	4.83 × 10^−5^	30	4.13	1	/	LSV	[23]
PVA/GA with 24 wt% of LiClO_4_	LiClO_4_	1.6 × 10^−4^	25	/	/	/	/	[24]
BCT (copolymer dblock)	LiTFSI	9.1 × 10^−6^	30	5	1	60	CV	[25]
5PEG-SSH	LiTFSI	7.28 × 10^−6^	30	5	10	60	LSV	[26]
Li(FSI-ethyl cellulose)/PEO	LiTFSI	0.5 × 10^−4^	70	4	10	70	LSV	[27]
Li-HCFu-PH	LiPF_6_	6.4 × 10^−3^	RT	4.7	1	RT	CV/ LSV	[28]
CPEG (copo EC/EO)	LiTFSI	1.84 × 10^−4^	30	4.75	1	60	LSV	[29]
(PEO)-based NASICON−LiZr_2_(PO_4_)	LiTFSI	1.2 × 10^−4^	30	5	/	/	LSV	[30]
Dual-Li SPEs	LiTFSI + LiPVFM	5.7 × 10^−4^	25	4.5	5	/	LSV	[31]
POSS-PEGDA/PEO/LiTFSI	LiTFSI	3.83 × 10^−4^	60	5.3	10	60	LSV	[32]
PVT-EMIMTFSI	EMIMTFSI	1.26 × 10^−4^	RT	4.5	10	25	LSV	[33]
PEO(LiTFSI)-LLZO+PEGDME	LiTFSI	4.7 × 10^−4^	60	5.2	1	60	LSV	[34]
PEO-SiO_2_	LiClO_4_	1.1 × 10^−4^	30	5	1	/	LSV	[35]
PEO/MnO_2_	LiTFSI	1.95 × 10^−5^	30	4.5	1	60	LSV	[36]
SIGPE	LiSTFSI	0.84 × 10^−3^	RT	5.2	1	RT	LSV	[37]
8-PEG-MALC, 8-PEG, PEGDA	LiTFSI	6.2 × 10^−5^	RT	4.5	1	RT	LSV	[38]
PEO/Al-MOF 5%	LiTFSI	2.09 × 10^−5^	30	4.7	5	30	LSV	[39]
PEO/MOF-UIO66	LiTFSI	1.47 × 10^−4^	30	5.2	0.5	/	LSV	[40]
PEO/PVDF/LiClO_4_/TiO_2_/PC	LiClO_4_	10.2 × 10^−6^	27	3	/	RT	LSV	[41]
polycarbonates/polyethers with linear and cyclic carbonates linkages	LiTFSI	5.6 × 10^−5^	25	5.6	0.5	70	CV	[42]
LiTFPFB/P(PO/EM)	LiTFPFB	1.55 × 10^−4^	70	4.6	5	70	LSV	[43]
CPE-(SiO_2_@PMMA)	LiTFSI	8.54 × 10^−5^	60	4.7	0.5	/	LSV	[44]
PEO/LDH (layer double hydroxide)	LiTFSI	1.1 × 10^−5^	30	5	0.5	/	LSV	[45]
CSE (PEO-LiClO_4_-PVDF/Al-LLZO)	LiClO_4_	1.73 × 10^−4^	70	5.25	2	70	LSV	[46]
PVDF-HFP-LLZO	LiTFSI	1.12 × 10^−3^	30	4.6 to 4.9	1	/	LSV	[47]
LAGP–PEO LAGP-PEA LAGP-epoxy	LiTFSI and LiCLO_4_	1.25 × 10^−4^ 7.4 × 10^−4^ 8.4 × 10^−4^	25 25 25	4.5	0.1	/	CV	[48]
CS(chitosan)-LiTFSI-PEO	LiTFSI	6.8 × 10^−4^	RT	5	5	/	LSV	[49]
LLTO-PAN-SN(succinonitrile)	LiTFSI	2.2 × 10^−3^	30	5.1	1	25	LSV	[50]
LSZP-PVDF	LiTFSI	5.76 × 10^−5^	25	4.73	0.2	RT	LSV	[51]
P(EGDMA-DODT)	LiTFSI	2.7 × 10^−5^	RT	4.3	/	25	CV	[52]
PVDF/PEO/LiClO_4_	LiClO_4_	2.01 × 10^−5^	27	3	50	RT	LSV	[53]
poly(ethylene oxide carbonate)	LiTFSI	1.2 × 10^−4^	70	4.9	0.5	70	LSV	[54]
HCPE (derived PEG SPE)	LiTFSI	5.62 × 10^−5^	RT	5.28	10	25	LSV	[55]
poly (PEGDA-PEMP-PDMS)	LiTFSI	1.08 × 10^−5^	25	5	1	60	LSV	[56]
Cross-linked nanoparticle-polymer composites (CNPCs) OH-PEO-SiO_2_	LiTFSI	3 × 10^−3^	25	5	1	RT	LSV/CV	[57]
PEO-LATP	LiTFSI	1.15 × 10^−5^	30	5	/	30	CV/LSV	[58]
PVAc in P(VdF-HFP)-LiTFSI-EC	LiTFSI	1.1 × 10^−3^	RT	4.7	0.5	RT	LSV	[59]
poly(PEGDGE-PEMP-PDMS)	LiTFSI	1.5 × 10^−6^	RT	5.3	0.1	60	CV	[60]
PEG250-POSS-4PEG2k	LiTFSI	3 × 10^−4^	RT	4	/	90	CV	[61]
PEO/LAGP	LiTFSI	1.6 × 10^−5^	20	4.5	0.1	60	CV	[62]
LLTO(NF)/PEO	LiClO_4_	4.01 × 10^−4^	60	5.1	1	60	LSV	[63]
PEO34-PC 10 wt% MA	LiTFSI	1.3 × 10^−3^	70	4.9	0.5	70	CV	[64]
P(SSPSILi-alt-MA)/PEO	SSPSILi	3.08 × 10^−4^	25	5	10	80	LSV	[65]
HSPE(polysiloxane/polyetherdiamine)	LiClO_4_	5.8 × 10^−4^	80	4.8	1	/	LSV	[66]
Grafted polyrotaxane	LiTFSI	1 × 10^−4^	RT	4.7	0.05	60	LSV	[67]
NOE/PEO and LSA/PEO	LiTFSI	5.08 × 10^−5^	RT	4.2	10	/	CV	[68]
PEO-CuO fillers	LiTFSI	1 × 10^−4^	30	4.8	1	25	LSV	[69]
bis(2-methoxyethyl) ether (diglyme)	LiNO_3_	/	/	4.5	10	RT	LSV	[70]
PEO-BaTiO_3_	LiTFSI	1.8 × 10^−5^ 1.6 × 10^−3^	25 80	4.7	0.5	80	LSV	[71]
PEGDGE	LiTFSI LiBF_4_	0.11 × 10^−3^	RT	5.5	0.1	60	LSV/CV	[72]
PEO/TDI/PEG	LiTFSI	0.17 × 10^−3^	60	5	0.5	60	LSV/CV	[73]
PIL-LiTFSI-LATP	LiTFSI	7.78 × 10^−5^	30	4.5	5	60	LSV	[74]
Anion-regulated PEGPEA-SiO_2_	LiTFSI	2.16 × 10^−5^	RT	4.8	0.1	55	CV	[75]
PEO@GF	LiTFSI	1.9 × 10^−4^	60	4.9	0.1	/	LSV	[76]
UV-PCCE	LiTFSI	0.91 × 10^−3^	RT	4.78	0.1	25	CV/LSV	[77]
(PTHF)-based SPE	LiClO_4_	2.3 × 10^−4^	60	4.5	1	60	CV	[78]
HGO(holey graphene oxide)-PEO	LiTFSI	6.05 × 10^−4^	60	5.2	5	/	LSV	[79]
PEO:LiTFSI:SN(15%):LAO(10%)	LiTFSI	1.36 × 10^−5^	30	5.2	10	60	LSV	[80]
PEO-LLZTO-MMT	LiTFSI	4.7 × 10^−3^	70	4.6	10	/	LSV	[81]
cross-linked-PEO-TEGDME-TEGDMA	LiTFSI	2.7 × 10^−4^	24	5.38	0.1	25	CV/LSV	[82]
PEO-BaTiO_3_	LiTFSI	2.2 × 10^−5^ 1.9 × 10^−3^	25 80	5	0.5	80	LSV	[83]
PVA, PMP-TFSI	LiTFSI	3 × 10^−3^	60	4.6	0.5	/	LSV	[84]
NH2-PEG-NH2	LiClO_4_, LiTFSI, LiBF_4_	1.9 × 10^−4^	RT	5	0.1	/	LSV	[85]
PEO grafted polyimide (PI-g-PEO)	LiTFSI	1 × 10^−4^	40	5	0.1	60	CV/LSV	[86]
LLZN NWs filled PMMA-LiClO_4_	LiClO_4_	2.2 × 10^−5^	RT	4.7	1	60	LSV	[87]
Halloysite nanotubes (HNTs)-PEO	LiTFSI	9.23 × 10^−5^	25	5.14	10	25	LSV	[88]
PVDF-HFP/PEO/LAGP	LiFSI	3.27 × 10^−3^	RT	4.9	1	/	LSV	[89]
POSS−PEG−PIL	LiTFSI	1.86 × 10^−5^ 2.07 × 10^−4^	25 60	4.7	1	90	LSV	[90]
vertically aligned LAGP- PEO	LiTFSI	1.67 × 10^−4^	RT	4.5	5	60	CV	[91]
PVDF-HFP/LiTFSI/LLZO	LiTFSI	9.5 × 10^−4^	RT	5.2	0.1	RT	LSV	[92]
PIL-PEO	LiTFSI	6.12 × 10^−4^	55	5.44	1	55	LSV	[93]
LLZO-PVDF	LiClO_4_	2.6 × 10^−4^	25	4.8	1	25	LSV	[94]
PVA-Upy-PEG750	LiClO_4_	1.51 × 10^−4^	60	5	0.1	RT	LSV	[95]
PS-PEG-PS	LiTFSI	1.1 × 10^−3^	70	4.5	0.1	70	CV/LSV	[96]
(PEO) K-SPE750-Li	LiClO_4_	2.82 × 10^−5^	20	5.4 5.3	0.1	60 25	LSV LSV	[97]
LiFPFSI/PEO	LiFPFSI	6.2 × 10^−4^	80	5.6	0.5	80	LSV	[16]
sPS-LiTFSI/PEGDA/succinonitrile	LiTFSI	0.43 × 10^−3^	RT	5–5.3	0.5	/	LSV/CV	[98]
PGO	LiClO_4_	2.08 × 10^−5^	50	4.4	1	RT	LSV	[99]
PPC-PEO 10 W [5:5]-1%wt LAGP	LiTFSI	8.39 × 10^−4^	60	4.5	0.5	60	LSV	[100]
PVDF/PVAC-LLZTO	LiClO4	4.8 × 10^−4^	RT	4.85	0.1	/	LSV	[101]
PEO-N1222FSI-LiFSI	LiFSI	2.14 × 10^−4^	50	5	1	50	LSV	[102]
Li-Nafion/LLZAO	/	2.26 × 10^−4^	30	4.8	0.1	30	CV/LSV	[103]
PPO-PEO-PPO/HO-PEO-SiO_2_	LiPF_6_/LiTFSI	1.32 × 10^−3^	20	6.5	1	20	CV	[104]
Sandwich-type PVDF-HFP-LLZTO	LiTFSI	2.29 × 10^−4^	30	5.3	1	40	LSV	[105]
LLZTO/PEO-LiTFSI	LiTFSI	2.61 × 10^−4^	25	6	1	/	LSV	[106]
PEO-ta-POSS	LiTFSI	1.2 × 10^−3^	90	3.8	0.2	/	CV	[107]
g-C3N4/PEO (CSPE)	LiTFSI	1.7 × 10^−5^	30	4.7	5	60	LSV	[108]
N1222FSI-PIL	LiTFSI	2.08 × 10^−4^	25	5	1	40	LSV	[109]
hbPPEGMAm-s-PSn	LiTFSI	9.5 × 10^−5^	60	4.3	0.2	/	LSV	[110]
PEO-cPTFBC	LiDFOB	2.2 × 10^−5^	50	4.7	1	60	LSV	[111]
PEO@SiO_2_	LiClO_4_	1.1 × 10^−4^	30	4.8	10	90	LSV	[112]
LLZTO/PEO	LiTFSI	1.31 × 10^−5^	25	5.2	1	RT	LSV	[113]
